# A usability design checklist for Mobile electronic data capturing forms: the validation process

**DOI:** 10.1186/s12911-018-0718-3

**Published:** 2019-01-09

**Authors:** Alice Mugisha, Victoria Nankabirwa, Thorkild Tylleskär, Ankica Babic

**Affiliations:** 1 Centre for International Health, University of Bergen, Bergen, Norway; 2 School of Computing and Informatics Technology, Makerere University, Kampala, Uganda; 3 School of Public Health, Makerere University, Kampala, Uganda; 4 Centre for Intervention Science in Maternal and Child Health (CISMAC), Centre for International health, Department of Global Public Health and Primary Health Care, Faculty of Medicine and Odontology, University of Bergen, Bergen, Norway; 5 Department of Information and Media Studies, University of Bergen, Bergen, Norway; 6 Department of Biomedical Engineering, Linköping University, Linköping, Sweden

**Keywords:** Mobile electronic data capturing forms (MEDCFs), Usability, Specific application domain (SAD) heuristics

## Abstract

**Background:**

New Specific Application Domain (SAD) heuristics or design principles are being developed to guide the design and evaluation of mobile applications in a bid to improve on the usability of these applications. This is because the existing heuristics are rather generic and are often unable to reveal a large number of mobile usability issues related to mobile specific interfaces and characteristics. Mobile Electronic Data Capturing Forms (MEDCFs) are one of such applications that are being used to collect health data particularly in hard to reach areas, but with a number of usability challenges especially when used in rural areas by semi literate users. Existing SAD design principles are often not used to evaluate mobile forms because their focus on features specific to data capture is minimal. In addition, some of these lists are extremely long rendering them difficult to use during the design and development of the mobile forms. The main aim of this study therefore was to generate a usability evaluation checklist that can be used to design and evaluate Mobile Electronic Data Capturing Forms in a bid to improve their usability. We also sought to compare the novice and expert developers’ views regarding usability criteria.

**Methods:**

We conducted a literature review in August 2016 using key words on articles and gray literature, and those with a focus on heuristics for mobile applications, user interface designs of mobile devices and web forms were eligible for review. The data bases included the ACM digital library, IEEE-Xplore and Google scholar. We had a total of 242 papers after removing duplicates and a total of 10 articles which met the criteria were finally reviewed. This review resulted in an initial usability evaluation checklist consisting of 125 questions that could be adopted for designing MEDCFs. The questions that handled the five main categories in data capture namely; form content, form layout, input type, error handling and form submission were considered. A validation study was conducted with both novice and expert developers using a validation tool in a bid to refine the checklist which was based on 5 criteria. The criteria for the validation *included utility, clarity, question naming, categorization and measurability*, with *utility* and *measurability* having a higher weight respectively. We then determined the proportion of participants who agreed (scored 4 or 5), disagreed (scored 1 or 2) and were neutral (scored 3) to a given criteria regarding a particular question for each of the experts and novice developers. Finally, we selected questions that had an average of 85% agreement (scored 4 or 5) across all the 5 criteria by both novice and expert developers. ‘Agreement’ stands for capturing the same views or sentiments about the perceived likeness of an evaluation question.

**Results:**

The validation study reduced the initial 125 usability evaluation questions to 30 evaluation questions with the form layout category having the majority questions. Results from the validation showed higher levels of affirmativeness from the expert developers compared to those of the novice developers across the different criteria; however the general trend of agreement on relevance of usability questions was similar across all the criteria for the developers. The evaluation questions that were being validated were found to be useful, clear, properly named and categorized, however the measurability of the questions was found not to be satisfactory by both sets of developers. The developers attached great importance to the use of appropriate language and to the visibility of the help function, but in addition expert developers felt that indication of mandatory and optional fields coupled with the use of device information like the Global Positioning System (GPS) was equally important. And for both sets of developers, utility had the highest scores while measurability scored least.

**Conclusion:**

The generated checklist indicated the design features the software developers found necessary to improve the usability of mobile electronic data collection tools. In the future, we thus propose to test the effectiveness of the measure for suitability and performance based on this generated checklist, and test it on the end users (data collectors) with a purpose of picking their design requirements. Continuous testing with the end users will help refine the checklist to include only that which is most important in improving the data collectors’ experience.

## Background

Over the years, electronic data collection systems are increasingly being used in health care particularly for data collection and management in health surveys, surveillance and patient monitoring [1]. Electronic data collection tools consist of mobile devices like phones, computers and tablets (hardware) together with a number of different possible programs (software), also known as form creation software [2] which maybe open-source or proprietary. For mobile electronic data collection systems, data collection is done using mobile forms, known as Mobile Electronic Data Collection Forms (MEDCFs), which are developed and designed by software developers and form developers respectively. The form developers do not need to have any prior software programming training, but rely on the array of tools provided by the software [2] to create the forms. These electronic forms usually consist of numeric fields and multiple choice menus, among others [3] and their main role is to collect data through direct data capture.

Usability is considered as one of the top attributes of assessing quality and its major role is to ensure that the interfaces are easy to use and that users are supported in performing their tasks efficiently and effectively [4]. One of the ways of ensuring usability is through performing Heuristic Evaluation on the interfaces, where “reviewers, preferably experts, compare a software product to a list of design principles (or heuristics) and identify where the product does not follow those principles” [5]. Heuristic evaluation is the most popular and commonly used usability inspection method because of its high benefit to cost ratio in cases where time and resources are scarce [6]. It is important to note however that these software products vary in functionality, design and features, and thus would require different design principles that are specific to their domain. For example Nielsen’s usability heuristics have been said to give inaccurate results for heuristic evaluations involving non-traditional types of software like transactional websites and mobile based applications among others [7]. This is because Nielsen’s are desktop-oriented heuristics and therefore may fail to reveal a large number of mobile usability issues related to mobile specific interfaces and characteristics [8, 9].

Heuristics that are applicable in one context may not work in another, or may sometimes contradict a heuristic used in another context. Secondly, their being broad often leaves room for the individual evaluator’s interpretation of what they mean, and may also be challenging to less experienced evaluators in finding pertinent design problems [5, 10]. There is therefore a need for more accurate inspections and assessment tools where evaluators can identify, beyond the generic usability problems, issues from the specific application domain [11, 12], hence a need for Specific Application Domain (SAD) heuristics.

The study therefore sought to generate and validate a design checklist for MEDCFs. We proposed a list of sub-heuristics from literature which focus on the data capturing process. We later validated this list in order to transform and refine it, so that it would be more usable to the intended users.

## Methods

To develop a design checklist for mobile electronic data collection forms (MEDCFs), we used different types of techniques and involved different stakeholders. First we conducted a literature review where we generated an initial usability evaluation checklist. The initial checklist was then validated by a team of software developers using a validation instrument.

### Literature review

We used the ACM digital library, Springer, IEEE-Xplore and Google scholar, together with some literature on best practices from other sources like Google. Our key terms in the search included ‘usability’, ‘usability evaluation’, ‘heuristics’, ‘mobile’, ‘tool’, ‘checklist’, ‘user interface’ and ‘design’. The key words were chosen with a focus on improving usability in mobile electronic data collection based on the assumption that usability can be improved through proper designing and evaluation of the user interfaces by developers using heuristics/design principles that are specific to MEDCFs. The inclusion criteria included papers that were focused on computer applications, usability, heuristic evaluation, generation and validation of heuristics.. Our search generated 242 papers for review after removing the duplicates. On screening based on titles, we then removed 17 papers whose titles did not have the words ‘*usability’, ‘evaluation’, ‘mobile’ and ‘heuristic’* and were left with 225 articles*.* We screened the abstracts and removed those papers which were not in English and those which were not about usability evaluation of user interfaces, leaving us with 134 articles. We then omitted those papers which were not focused on generating or validating usability heuristics for mobile devices or interfaces, and we were then left with 10 articles.

### Generating the initial usability evaluation checklist

We derived our usability evaluation questions from 10 papers (Thitichaimongkhol and Senivongse. 2016, Gomez et al. 2014, Omar et al. 2016, Nielsen 2001b, Pierotti 1995, Budiu and Nielsen 2011, of Health and Services nd, Parham 2013, Nielsen 2001a, Nayebi et al. 2013), the majority of which came from a system checklist by Pierotti [13]. Other sub heuristics were also derived from the ERP checklist, one of the latest mobile based checklists and also an update of the usability heuristic checklist for mobile interfaces [14]. We therefore updated this checklist by removing some evaluation questions that are specific to mobile ERP and were then left with 125 usability evaluation questions. These questions were derived from sub-heuristics for mobile applications coupled with those from a number of usability heuristic studies and usability guidelines for online web forms [15, 16].

### Categorization and rearranging of the selected sub-heuristics

We selected those sub-heuristics that fitted in the 5 categories and are representative of the data capturing process as shown in the design of web forms. This included transfer of sub heuristics from their original sub-heuristic category and placed under a new one based on what they are evaluating. The categories comprised of the *form content, form layout, input process, error handling* and *form submission* [15]. We then merged the mobile sub heuristics with some of the web form usability guidelines. We however changed the ‘input type’ to ‘input process’ because the input type only relates to how data should be entered into the form [15], and yet we sought to evaluate the data collection forms beyond just inputting data, but including other features that may influence the input process like the *visual feedback* and *list pickers* among others. The 125 questions were categorized as follows. *Form content* had a total of 35 questions, *form layout* had a total of 43 questions and the *input process* category comprised of 22 questions. *Error handling* had 23 questions, while *form submission* had the least with only 2 questions.

#### Form content

The form content depends on the data being collected. Some forms may be in form of questionnaires, whereas others may be in tabular form, hence the variation in content. The main content is usually questions and labels or fields of text entry. However, it is very crucial to map the environment which the users are familiar with in order to ease the use of the form. In this case, designing an electronic form that is analogous to the paper forms will quicken the data collectors’ understanding of the form [17].

#### Form layout

The form layout shows how the form is presented on the mobile user interface, and this influences the way a user interacts with it. The form layout is still determined by the nature of the content that is being collected. For example long survey questionnaires will have a different layout from a short mostly graphical form used by a clinician in a health facility. In addition, the designs, positions and lengths of the labels and input fields, the date format, number of columns and buttons among others all define the layout of the form [17].

#### The input type

This refers to the way data is captured or entered into the form and therefore which input type is most appropriate for a given case for example check boxes, radio buttons etc. Care should always be taken not to confuse users by using many different input types in one form [18]. In some instances, frequent use of text boxes is recommended [19], but not in cases where the number of possible answers has to be limited [20] because then radio buttons, check boxes or dropdown menus can be used comfortably. On the other hand, the use of text boxes can contribute to typing errors and delays in data collection more than when users have to select from a given set of options. The input process can also be determined by the type of analysis one is going to perform or on the decisions to be made with the collected data.

#### Error handling

Users need to be guided as quickly and as error free as possible during the process of filling forms from the start by explaining restrictions in advance [17]. This includes formatting and content rules such as minimum length of numbers or words, entry formats, putting help instructions, etc. being communicated well in advance. There are various ways of communicating e.g. by indicating the format specification where a user cannot miss it for example inside the text box. Sometimes errors are unavoidable, and therefore users need to be helped to recover from them as quickly and as easily as possible by clearly stating what the error is and how it can be corrected in a familiar language [20, 21].

#### Form submission

The form has to be submitted after filling it using a submission button [17]. The submission button needs to be disabled after the first submission to avoid multiple submissions in cases of system response delays [20]. The positioning of the reset or cancel button should also be carefully considered or the button avoided as it can lead to a cancellation of the already completed work accidentally. After submission of the form, the recipients need to acknowledge receipt of the form [20, 22].

We chose to use these categories because they represent some of the main activities a user is involved in when filling a data collection form. And therefore were a good basis for the selection and categorization of the questions that we included in the initial usability evaluation checklist.

### Validation of the derived usability evaluation questions by the software developers

Heuristics for Specific Application Domains (SAD) can be generated in a number of ways, but one of the most important steps is the validation of the heuristics to ensure that they are able to do what they are supposed to do. According to Van Greunen et al., [23] the validation phase is the second of three phases in the 3-phase process to develop SAD heuristics and it consists of 4 major tasks. These include; identification and selection of experts who have the theoretical knowledge and practical experience with regards to SAD. The second task is the application of the validation tool to assess the heuristics using rating scales to measure their characteristics these characteristics are likely to have an impact on the adoption of the new heuristics for the SAD. The third task is to analyze the results from the validation process in order to determine the necessary modifications to the heuristics. The last task involves iterating and redesigning the heuristics until the experts are satisfied with the outcome [23].

The software developers validated this initial usability evaluation set in order to refine it further and make it more usable using a validation tool. The validation tool was created as an excel file and each of the 125 usability evaluation questions was scored on a scale of 5 where the options were presented in form of a drop down list. These included *strongly disagree, disagree, somewhat agree, agree* and *strongly agree* with a score of 1,2,3,4 and 5 respectively. Furthermore, the developers were free to add a comment explaining their scores in addition to removing or adding to the usability evaluation questions. The developers could also suggest renaming a given question or re-locating a question to a different category.

The validation tool was based on 5 major assessment criteria, namely utility, clarity, question naming, categorization and measurability. The criteria was based on characteristics proposed by Van Greunen et al. [23] some of which included naming and importance of high level heuristics, grouping of checklist items under heuristic names and ease of use. Because utility is a part of usefulness, it also qualified as part of the assessment criteria [24]. We also considered measurability because it is important that the heuristics are quantifiable in order to rate them appropriately. Utility and measurability are considered to have a higher weight because the utility or measurability of a heuristic during evaluation cannot be compromised otherwise it would not be suitable for inclusion in the checklist. Other criteria that could have been considered in this study included thoroughness, reliability, effectiveness, cost effectiveness and validity [25]. However, these would be beneficial in assessing complete heuristics in real work contexts, and thus would not be very feasible in our contexts.

#### Utility

This tests the evaluation question’s contribution and relevance to the design of the mobile data collection form.

#### Clarity

This tests whether the evaluation question is clear and can easily be understood by the evaluator.

#### Question naming

The test was on whether the evaluation question name was appropriate.

#### Categorization

Here the test was whether the evaluation question is placed in the right category.

#### Measurability

This tested the possibility of measuring and attaching a score to the design feature using this evaluation question.

Validation can be a continuous and iterative process involving novice, average and expert users. The initial assessment of the initial usability evaluation questions was performed by novice software developers in March 2017. We presented the main study objectives and the relevance of the activity the novice developers were about to undertake, after which they downloaded the checklist and the validation tool from their individual email addresses. We then trained the developers for about 10 min, after which they were given 90 min to assess the checklist and submit the completed assessment thereafter to the researchers’ email address.

The second validation of the same evaluation questions was done by the expert developers in Uganda between October and November 2017. The expert developers had developed mobile forms for collection of health data for varying periods of time, ranging from 1 year to 8 years using software like Open Data Kit (ODK) (*n* = 9), District Health Information Software (DHIS2) (*n* = 6), Open Medical Records System (OpenMRS) (*n* = 5). Other applications included mUzima (*n* = 2), Medic Mobile Toolkit, CSpro, Survey CTO, koBo Toolbox, Survey Monkey and OpenXData. The 20 developers received an email each indicating the main study objective and the relevance of the activity they were about to engage in. On acceptance to be part of the study, the file with the assessment criteria was forwarded to them via email, and they were expected to submit it after one week.

## Results

### Validation of the initial usability checklist by the novice developers

Out of the 20 copies of validation tools that were sent out, we received 18 copies back, 3 of which were incomplete. So our results were based on the 15 complete submissions. We received a total of 9 comments from 5 developers. Five of the comments mentioned that some of the evaluation questions were not clear and therefore could possibly lead to misinterpretation or confusion. For example two developers felt that question 12 *(Is the number of colors limited to 3–4?)* was unclear and one had to read it twice to understand it. Three of the comments went on to advise on how we could improve on a given question e.g. splitting question 9 *(Is only and all information essential to decision making displayed on the screen)* into 2 segments. One developer also felt that progress disclosure in question 38 *(Is there a link to each of the individual pages rather than just to the previous and next ones?)* would not be very important in a mobile view. All the 125 questions that were validated are listed in the Appendix.

### Validation of the initial usability checklist by the expert developers

All the 20 copies that were sent out to the expert developers were filled and received back after periods ranging from 2 to 4 weeks. We received more comments from the expert developers compared to the novice developers, some of which included renaming or re-categorizing the evaluation questions. For example some of the expert developers felt that questions 6, 10, 14, 15, 27, 28 and 29 needed to be put in other categories rather than the ones they were in (Appendix). In addition some of the questions were found to be inappropriate for this study e.g. (*Are all abbreviated words of the same length?).* Eight of the expert developers commented that it was not possible to have abbreviated words of the same length. Some questions were also found to be ambiguous e.g. (*Does the tool provide informative progress disclosure when filling a form?)* and *(Has the skip logic been automated?).* The relevance of some of the questions was also questionable e.g. *(Does the mobile tool’s UI keep the total number of touchable UI elements to less than 10 per view?)*. Some expert developers also pointed out that some of the design features’ performance is influenced by other factors e.g. the feedback time during data collection may be influenced by the internet speed. However this activity is rather subjective and very individualistic and therefore the developers were likely to interpret and evaluate the questions differently based on their varied experiences.

To generate the usability design checklist, we considered questions where 80% and above of the novice and expert developers agreed to each of the utility, clarity, question name, categorization and measurability of the questions. We then selected those questions where more than 80% of the responses indicated ‘agree’ or ‘strongly agree’ across all the 5 criteria. Because utility and measurability have higher weight than the rest of the criteria, we also considered those questions which scored above 80% in both usability and measurability. We then considered those questions where both novice and expert developers affirmed to the utility of the question. And lastly, we considered those questions where only the experts affirmed to the utility of the question. This led to a total of 64 questions. We then calculated the average of responses with ‘agree’ or ‘strongly agree’ for each question across the 5 criteria, and selected those questions with an average of 85% and above. This led to 30 evaluation questions of which 9 were categorized under the form layout, 12 under form content, 2 under the input process, 6 under error handling and 1 under form submission. These 30 usability evaluation questions are all represented in Table 1.

**Table 1:** Usability evaluation checklist from the novice and expert developers’ evaluation with questions that both novel and experienced developers estimated as highly relevant depicted by criteria scores of ‘4’ or ‘5’

No.	Usability evaluation question	Agreed %
1.	Is it possible to get a summary of all the data the user has entered at any given time?	94
2.	Are there visual differences between interaction objects (e.g., buttons) and information objects (e.g. labels, images)	94
3.	Are the data entry fields which are mandatory or required clearly marked?	94
4.	Does the tool make use of device information like data and time, geo-location, device number, etc. as input data?	94
5.	Do data entry screens and dialog boxes indicate when fields are optional?	93
6.	Does the tool show error signals and marks on the actual field that has an error and needs to be changed?	92
7.	Is there some form of feedback for every user interaction?	92
8.	Are the buttons in the form mostly or always visible?	90
9.	Is the submit button disabled as soon as it has been clicked during submission of the form?	90
10.	Is the help function visible?	90
11.	Does the tool preserve the user’s work in order to correct errors by just editing their original action instead of having to do everything over again?	90
12.	Can users easily switch between help and their work?	89
13.	Can users move forward and backward between text fields or dialog box options?	88
14.	Is the language used in the form clear, effective and appropriate for the target users?	89
15.	Is navigation consistent across orientations?	88
16.	Does the tool provide the user an alternate method of authentication?	88
17.	Does a back button simply return the form to a previous view without loss of data?	87
18.	For data entry screens with many fields can users save a partially filled form?	87
19.	Are users able to interact with the form by swiping or pinching (zooming in and out) instead of only touching?	87
20.	Is all the information users enter into the data forms validated and users informed if it is not in an acceptable format?	87
21.	Are inactive menu items greyed out or omitted?	87
22.	If pop-up windows are used to display error messages, do they allow the user to see the field in error?	87
23.	Are prompts, cues, and messages placed where the eye is likely to be looking on the screen?	87
24.	Is it possible to automatically save a page in the form when a user scrolls to the next page?	87
25.	Does the system provide an example input for format-specific or complex information?	87
26.	Is the format of a data entry value for similar data types consistent from screen to screen of a given form?	86
27.	Is the user able to know where he or she is during navigation of the form?	85
28.	Can users resume work where they left off after accessing help?	85
29.	Have the forms been designed to recognize specific input types and adjust the input modes accordingly during data entry?	85
30.	Users dislike typing, is information computed for the users where applicable?	85

There were no questions where both sets of developers selected ‘agree’ to all the 5 criteria for a particular question. However, there were 11 questions in this checklist where both sets of developers selected ‘agree’ to more than one criterion for a given question. But generally expert developers affirmed to the questions based on the given criteria compared to the novice developers.

We further analyzed the data based on the criteria to determine the participants’ decision for each usability evaluation question. We determined the number and the respective percentage of participants who agreed, disagreed and were neutral to a given criteria for a particular usability question for each of the experts and the novice developers.

For 25 usability evaluation questions 85% and above of the novice developers selected ‘agree’ i.e. *utility* had 12 questions (*8, 15, 22, 23, 27, 33, 58, 59, 90, 99, 114 and 120), clarity* had 8 questions *(6, 27, 34, 58, 59, 66, 81 and 84),* and *question naming* had 3 questions *(33, 34 and 36).* In addition, categorization had 6 questions *(6, 16, 21, 42, 58 and 59)* while measurability had 4 questions *(20, 37, 47 and 64)*. We also had about 12 novice developers (80%) selecting ‘agree’ to the utility of 14 questions, to the clarity of 24 questions, to the question names of 13 questions, to the categorization of 15 questions and to the measurability of 5 questions. These results depict that majority of the questions that the novice developers agreed to were clear to them. In fact all the novice developers agreed to the clarity of question 66 *(Is it possible to automatically save a page in the form when a user scrolls to the next page?)*. The number of novice developers who selected ‘disagree’ against questions was relatively low with the highest being 8 developers disagreeing with the question name for question 9 *(Is only and all information essential to decision making displayed on the screen?).* There were also 40 and 27% of the developers disagreeing with the clarity and the categorization of this question respectively. The biggest percentage of disagreements (above 27%) was made up of measurability (14 questions) followed by utility (11 questions) and clarity (11 questions), and yet these are the criteria with the highest weights.

There were 50 evaluation questions where 85% and above of the expert developers selected ‘agree’ for all the criteria apart from, *measurability* which was below 85%. There were incidences where all the expert developers affirmed to the criteria regarding a particular question for example utility had 5 questions *(28, 35, 45 92 and 119),* clarity had 5 questions *(27, 28, 34, 40 and 92)*, question name had 3 questions (15, 27, and 33) while categorization had 4 questions *(65, 92, 100 and 103).* Question 92 *(Does the tool make use of device information like data and time, geo-location, device number, etc as input data?)* however had all the expert developers agree to the utility, clarity and categorization of that question. In addition we had 7 questions *(23, 25, 33, 34, 35, 92 and 102)* where 90% and above of the expert developers agreed on the relevance of 3 criteria and 9 questions *(8, 15, 27, 28, 53, 65, 103, 119 and 124)* where 90% and above of the developers agreed on the relevance of the 4 criteria. This can be compared to questions 58 *(Are inactive menu items greyed out or omitted?)* and 59 *(Are prompts, cues, and messages placed where the eye is likely to be looking on the screen?)* where 87% of the novice developers agreed to the utility, clarity and categorization value of the questions.

We also considered those questions where less than 50% of the novice developers selected ‘disagree’ Measurability had 29 questions, followed by utility with 13, clarity with 9, question name with 8 and lastly categorization with 8 questions. Question 24 *(Are all abbreviated words of the same length)* had a high level of disagreement across all the 5 criteria, with utility having the highest diagreement of 50%.

**Fig. 1:**
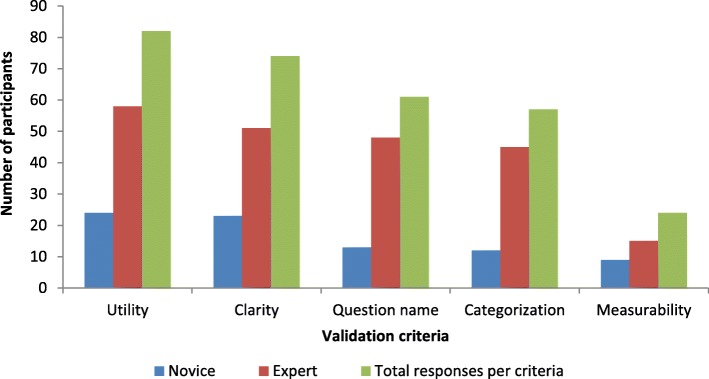
Total number of responses per usability criteria

The number of expert developers who gave high criteria scores for each usability question was higher than the number of novice developers. In addition, the scores across criteria also varied with the highest being utility followed by clarity, question name, categorization and lastly measurability, and for both sets of developers, utility scored highly while measurability scored least (Fig. 1).

## Discussion

### Principal findings

The literature search generated 125 usability evaluation questions which after validation by the novice and software developers were reduced to 30 questions. The results after the validation indicate that expert developers appeared to value the utility, clarity, question names, categorization and measurability of the questions more homogeneously as a group than the novice developers. According to both groups of developers, the questions were found to be useful, clear, with proper names and correct categorization; however both sets of developers felt that the measurability of the questions was not satisfactory.

The results indicate differences in the agreement and disagreement levels with evaluation criteria of the novice and expert developers, but the general trend was the same i.e. there were some questions where both evaluator groups agreed or disagreed. For example there were 20 questions where more than 85% of the developers found 3 or 4 criteria valuable. In addition, novice developers attached great importance to the use of appropriate language, omission of inactive menus, visibility of help function, prompts and messages and lastly to the ability to switch between data collection and their work. On the other hand, expert developers attached great importance to the appropriate language use as well, visual differences between interaction objects and information objects, indication of mandatory and optional fields, visibility of the help function and the use of device information like the Global Positioning System (GPS).

The variations in the levels of agreement and preferences may be attributed to differences in experience and context of operation. For example the expert developers had experience ranging from 3 to 10 years, which the novice developers did not have. Secondly the expert developers had been involved in creating MDCEFs for collection of health data in low resource settings, whereas the novice developers were more involved in mobile software development with nothing specific to Mobile data collection tools. However, in evaluation or validation it is important to have a mix of users with different skill levels e.g. the beginners or novice users, the average level users and the power or expert users to test a given product. This prevents skewing of the design requirements towards a particular group because users have varying needs based on their experience [26].

Most of the questions the developers affirmed to were in the form content category followed by the form layout, error handling, input process and the form submission categories respectively. Measurability was hardest to assess, followed by utility and clarity for both groups of software developers. This may be attributed to the fact that it was not clear to the developers what the criterion was meant to achieve. However, we still need to define ways of measuring the achievement of a particular design feature we are testing.

Expert developers had different views on more usability questions compared to the novice developers. This may be attributed to the experience they have had working on these applications such that they are able to know what is achievable or not. Secondly, some of the expert developers have had an opportunity of interacting and getting feedback from the end users especially during the training sessions, which placed them in a more advantaged position of knowing what the users may be comfortable with.

Utility of a usability question was considered most important among the validation criteria and as such, the evaluation questions with high percentages of disagreement for utility were automatically eliminated from the checklist. We argue that however clear or well categorized a usability question is, it needs to be useful in the evaluation of the MEDCFs.

### Limitations

One of the limitations was the difference in the time allocated to the 2 groups of developers; the expert developers had a lot more time to validate the initial checklist compared to the novice developers who only had 90 min. However, to the best of our knowledge, this was the first exercise of its kind where intended users get to validate the tool they will use to assess their interface designs for MEDCFs, besides the user testing of the prototype or finished product that is done with the end users. Having 2 sets of developers with varying experiences and from different contexts enriched the study because software developers only develop software with a focus on particular features which is partly the reason for the weaknesses of using Open Source Software (OSS) [27].

## Conclusion

The objective of this study was to compare the novice and expert developers’ views regarding usability criteria. This study generated and validated a design checklist for Mobile Electronic Data Capture Forms (MEDCFs), and was thus a way of creating awareness to what should be expected of a mobile data collection tool from the software developers’ perspective. 

The different results from the novice and expert developers, where we registered more affirmative results from the expert developers is an indicator of their expectations as developers. This may also be an indication of their level of engagement and knowledge of the people for whom they are creating the tools as well as the experience acquired over the years. We would thus recommend the use of more experienced developers during validation of checklists for mobile data collection tools.

The checklist resulting from this study needs to be evaluated by users as software developers are not the end users of the data collection forms. We thus propose to test the effectiveness of the measure for suitability and performance based on this generated checklist, and test it on the end users (data collectors) with a purpose of picking their design requirements. Continuous testing with the end users will help refine the checklist to include only that which is most important in improving the data collectors’ experience. In addition to this first study that summarized the observations, there will be a deeper data analysis based on the collected material to determine the relationships between the criteria scores on the evaluation checklist and the developer groups.

## Appendix

### Initial usability evaluation checklist

**Table 2:** Form content

1. Is there some form of feedback for every user interaction? [13, 28] 2. Is this feedback noticeable and readable? 3. Is this feedback given within a reasonable amount of time? [29] 4. Does the tool provide informative progress disclosure when filling a form e.g. percentage of completion or time to wait to complete the form? [5, 28] 5. After users complete a task or group of tasks does the feedback indicate that they can proceed to the next task? [13] 6. Are the icons used in the tool concrete and familiar? [13] 7. In the event that shapes are used as a visual cue in the tool, do they match the cultural conventions? [13] 8. Is the language used in the form clear, effective and appropriate for the target users? [14, 28] 9. Is only and all information essential to decision making displayed on the screen? [13, 28] 10. Is colour coding used for clarity where appropriate? [28] 11. Do the selected colours used in the form correspond to common expectations about colour codes? [13] 12. Is the number of colours limited to 3–4? [28] 13. Are different presentations adopted for each of the headings, subheadings and instructions? 14. Do the information elements e.g. images and labels stand out from the form background? [5] 15. Are there visual differences between interaction objects (e.g., buttons) and information objects (e.g., labels, images) [5] 16. Can the questionnaire be broken down into sections? 17. Can each section have a section name with a small introduction? 18. Are the rows and columns of a table designed to be clear and understandable by the users? 19. If the form has multipage data entry screens, do all pages have the same title? [13, 28] 20. Do help instructions appear in a consistent location across all the form screens? [13] 21. Is there a consistent icon design scheme and stylistic treatment across the form? [5, 13] 22. Is there consistent location of the menu across the form? [5] 23. Is all the information users enter into the data forms validated and users informed if it is not in an acceptable format? [28] 24. Are all abbreviated words of the same length? [13] 25. Is the format of a data entry value for similar data types consistent from screen to screen of a given form? [13] 26. Is the design on the input type e.g. text box or drop down consistent across the form? [5] 27. Do data entry screens and dialog boxes indicate when fields are optional? [13] 28. Are the mandatory or required data entry fields clearly marked? [5, 28] 29. Is the length of the page controlled? E.g. by limiting the number of questions on the page [13, 28] 30. Has the skip logic been automated? 31. Are the help instructions visually distinct and accessible? [13, 28] 32. If menu items are ambiguous, does the tool provide additional explanatory information when an item is selected? [13] 33. Is the help function visible; for example, a key labelled HELP or a special menu? [13, 14] 34. Can users easily switch between help and their work? [13, 28] 35. Can users resume work where they left off after accessing help? [13, 28]	

**Table 3:** The form layout

36. In instances where a form has many pages, is each page of the form labelled to show its relation to others? [13] 37. Is pagination shown at the bottom for those forms with several pages? [14] 38. Is there a link to each of the individual pages rather than just to the previous and next ones? [14] 39. For longer forms with multiple content sections, is there a short and clickable list of the sections at the top of the page? [13] 40. Are the buttons in the form e.g. the back button and the forward button mostly or always visible? [5] 41. Have the buttons on the form been designed in different sizes and colours to emphasize importance? [30] 42. Are users able to know where they are during navigation of the form? [28] 43. Is the main navigation menu placed in the left panel of the tablet or phone UI? [31] 44. Is the navigation regulated to ensure users do not have to navigate much? [14] 45. Can users move forward and backward between text fields or dialog box options? [13] 46. If the form has many pages, can users move backward and forward among all the pages in the set? [13] 47. If the tool uses a question and answer interface, can users go back to previous questions or skip forward to later questions? [13] 48. Are cancels/exits from pages or sections clearly marked? [14, 28] 49. Is it possible for users to undo their navigation in case they are not where they want to be? [14, 28] 50. Is there some level of personalization on the screen? [13, 28] e.g. on font sizes, viewing style 51. Is it possible to customize the error message in cases where users fail to understand the questions egg changing the language 52. Are users able to change the orientation of the form during data capture? [5, 14, 28, 32] 53. Is navigation i.e. horizontal or vertical consistent across orientations? [14, 32] 54. Is content consistent across orientations? [5, 14, 28, 32] 55. Are menu choice lists presented vertically? [13] 56. If “exit” is a menu choice, does it always appear at the bottom of the list? [13] 57. Are menu titles either centred or left-justified? [13] 58. Are inactive menu items greyed out or omitted? [13] 59. Are prompts, cues, and messages placed where the eye is likely to be looking on the screen? [13] 60. Do text areas have “breathing space” around them? [13] 61. Are size, boldface, underlining, colour, shading, or typography used to show relative quantity or importance of different screen items? [13] 62. Is there good colour and brightness contrast between image and background colours? [13] 63. Is the respondent able to add, remove or update their responses in the form as and when the respondent feels the need to? 64. Is the data the users enter into the form saved automatically such that they only have to save when necessary? [28] 65. Is it possible to get a summary of all the data users have entered at any given time? 66. Are there shortcuts in case one needs to back track? 67. Are users able to interact with the form by swiping or pinching (zooming in and out) instead of only touching? [5] 68. Is layout clearly designed avoiding visual noise? [14] 69. Are meaningful sections of questions separated by white space? [13] 70. Is it possible to see all the questions in one view without scrolling? 71. Does the mobile tool’s UI keep the total number of touchable UI elements to less than 10 per view? [30] 72. Is the number of submissions and clicks minimized during the process of entering data into the form? [14, 32] 73. If users are working from hard copy, does the screen layout match the paper form? [13] 74. Authorization and authentication. 75. If the tool does not store any information that is sensitive are users kept logged in but with an option of logging out when necessary? [14, 32] 76. When logging in must be done, is there an option that allows users to see the password clearly? [14, 32] 77. Does the tool provide the user an alternate method of authentication? 78. Does the tool help users to retrieve the login data in case they have forgotten? [33]	

**Table 4:** The input process

79. Is it possible to see a single response that has been selected in the form when surrounded by unselected options? [13] 80. Is there visual feedback in menus or dialog boxes about which response choices are selectable? [13] 81. Is there visual feedback in menus or dialog boxes about which choice the cursor is on at any given time? [13] 82. If multiple options can be selected in a menu or dialog box, is there visual feedback about which options are already selected? [13] 83. Is there a visible clue that shows users that they can swipe across the user interface? [14] 84. Are the list pickers e.g. drop downs more frequently used during data capture than text fields? [28] 85. Are data entry or text fields large enough to show all the entered data without scrolling? [31] 86. Can users reduce data entry time by copying and modifying existing data? [13] 87. Are character edits allowed in data entry fields? [13] 88. If menu lists are long e.g. more than 7 items on the response choice menu, can users select an item, either by scrolling or by typing a mnemonic code (filtering)? [5, 13] 89. Are field labels close to fields, but separated by at least one space? [13] 90. Are multiword field labels placed horizontally and not stacked vertically? [13] 91. When users enter a screen or dialog box, is the cursor already positioned in the field users are most likely to need? [13] 92. Has auto-tabbing been avoided except when fields have fixed lengths or users are experienced? [13] 93. Users dislike typing, is information computed for them where applicable? [14, 32] e.g. Age 94. Does the tool make use of device information like data and time, geo-location, device number, etc. as input data? [5] 95. Does the tool automatically align format for numeric values e.g. entering currency symbol, entering commas in numeric in numeric values greater than 9999? [13] 96. Do field labels appear to the left of single fields and above list fields? [13] 97. Are field labels and fields distinguished typographically? [13] 98. Is there consistent design on input element (e.g., textbox, dropdown)? [5] 99. Is the input element style modified too much? Can users recognize how to interact with the element? [5] 100. If expandable menus are used, do the menu labels clearly indicate that they expand to a set of options? [5]	

**Table 5:** Error handling

101. If pop-up windows are used to display error messages, do they allow users to see the field in error? [13] 102. Does the tool show error signals and marks on the actual field that has an error and needs to be changed? [5] 103. Are users prompted to confirm commands that have drastic, destructive consequences? [13, 34] e.g. deleting the form 104. Is there an “undo” or “redo” function during data entry in the form or after completing a task or group of tasks? [14, 28] 105. Are users able to leave an unwanted state without having to embark on an unwanted user interface interaction? [28] 106. Does the tool warn users if they are about to make a potentially serious error? [13] 107. Does the tool prevent users from making errors whenever possible? [13] 108. Do data entry fields and dialog boxes indicate the number of character spaces available in a field? [13] 109. Do fields in data entry screens and dialog boxes contain default values when appropriate? [13] 110. Is the data specific format or input type expected of the respondent indicated where applicable before they attempt to enter text in a given field? 111. Are the data format requirements put inside or outside of the text box? 112. On the form, is the location of positive button (e.g., OK button, next button) on the right and negative button (e.g., cancel button, back button) on the left? [5, 13] 113. Are touchable areas sufficiently big? [13, 32] 114. Are the touchable objects e.g. buttons in the screen placed too close? [5] 115. Is crowding targets avoided? For example when targets are placed too close to each other, users can easily hit the wrong one [13, 32] 116. Are the data input types appropriate for the type of information being entered in the field e.g. use number input type for numeric information [5] 117. Although the visible part of the target may be small, is there some invisible target space that if users hit that space, their tap will still count? [13, 32] 118. When signalling an input error in a form, is the field that needs to be changed specifically marked? [14, 32] 119. Does the tool preserve users’ work in order to correct errors by just editing their original action instead of having to do everything over again? [35] 120. Does a back button simply return the form to a previous view without loss of data? [28] 121. Does the tool reduce the work of correcting the error? Does it guess the correct action and let users pick it from a small list of fixes? [21] 122. If an error is detected, does the tool tell the user what happened, why and how to fix it? [28] 123. Does the system provide an example input for format-specific or complex information? [5]	

**Table 6:** Form submission

124. For data entry screens with many fields can users save a partially filled form? [13] 125. Is the submit button disabled as soon as it has been clicked during submission of the form?	

## Abbreviations

DHIS: District Health Information Software
GPS: Global Positioning System
MEDCFs: Mobile Electronic Data Capturing Forms
ODK: Open Data Kit
Open MRS: Open Medical Records System.
OSS: Open Source Software
SAD: Specific Application Domain
